# Job crafting, positive psychological capital, and social support as predictors of job embeddedness on among clinical nurses- a structural model design

**DOI:** 10.1186/s12912-024-01845-9

**Published:** 2024-03-22

**Authors:** Mi-Soon Yun, Miyoung Lee, Eun-Hi Choi

**Affiliations:** 1 Department of Nursing, Andong Science College, 36616 Andong, Republic of Korea; 2 Department of Nursing, Woosong University, 34606 Daejeon, Republic of Korea; 3 College of Nursing, Eulji University, 11759 Uijeongbu, Republic of Korea

**Keywords:** Role conflict, Positive psychological capital, Social support, Job crafting, Embeddedness

## Abstract

**Background:**

This study establishes the relationships among role conflict, positive psychological capital, social support, job crafting, and job embeddedness among clinical nurses. The results are expected to provide a basis for efficient human resource management in hospitals.

**Methods:**

Considering a 15% dropout rate, we distributed 300 copies of our questionnaire, of which 290 were returned. We used 260 responses in the final analysis after excluding 40 responses that were incomplete or showed an identical pattern in the item responses. Participants were clinical nurses with less than one year of experience in general and tertiary general hospitals in G province and D metropolitan city in South Korea. A structured questionnaire was administered from January 10 to February 28, 2022. The collected data were analyzed using SPSS 26.0 and AMOS 26.0. We assessed the statistical significance using the bootstrapping method.

**Results:**

The direct and total effects (both β = 0.806, *p* =.007) of positive psychological capital on job crafting were significant. The direct and total effects (both β = 0.451, *p* =.004) of social support on job crafting were significant. The direct (γ = 0.292, *p* =.055), indirect (γ = -. 671, *p* =.003), and total (γ = − 0.379, *p* =.008) effects of role conflict on job crafting were significant. The direct (γ = − 0.382, *p* =.007), indirect (γ = − 0.208 *p* =.003), and total (γ = − 0.589, *p* =.006) effects of role conflict on job embeddedness were significant. The direct and total (both β = 0.548, *p* =.005) effects of job crafting on job embeddedness were significant.

**Conclusions:**

Nurses’ job embeddedness is directly influenced by their job crafting, which is shaped by high levels of positive psychological capital and social support. When job crafting takes place, role conflict increases, and if job crafting becomes difficult because of severe role conflict, job embeddedness decreases. Therefore, to increase job embeddedness among clinical nurses, hospitals must implement support systems and programs to increase job autonomy, and positive psychological capital to promote job crafting.

## Background

Nurses, as the key human resources in hospitals, play the crucial role of providing close-contact nursing care to patients [1]. As the nursing role is becoming professionalized, it is increasingly important not only to have an adequate number of nurses but also to ensure the quality of those nurses [2]. However, nurses have high turnover intention. According to the Hospital Nurses Association’s staffing status survey, nurses had turnover rates of 15.4% in 2019 and 14.5% in 2021, with 17.1% citing “job inadequacy” as the reason for leaving [3]. The Korea Health and Medical Workers’ Union reported that 80% or more of nurses with 3‒10 years of experience have considered leaving in the past 3 months [4].

Nurse turnover leads to a shortage of experienced nurses, which increases the workload of their remaining co-workers, leading to job dissatisfaction and a vicious cycle of nurse turnover [5], which has a negative impact on the safety of patients and organizational performance of the hospital [6]. Therefore, recruiting and managing human resources has emerged as a challenge for hospitals to improve their competitiveness and organizational efficiency, of which hiring adequate nursing staff and retaining experienced nurses are important factors [7].

Substantial research has been conducted on reducing turnover intention among nurses, but the reasons for leaving have proven difficult to identify [8]. Research has examined nurses’ retention intention, or their intention to stay in their current jobs rather than seek other employment [9]. Recent studies have focused on why nurses stay at their hospitals [10]. Against this backdrop, the concept of job embeddedness has emerged as an influencing factor that keeps individuals at their places of work, going beyond the intention to stay in an organization [11]. Embeddedness refers to the quality of being firmly and deeply ingrained or fixed in place, and job embeddedness has developed as a concept to elucidate why individuals stay with an organization even when they are not satisfied with their job or relationship with it—that is, job embeddedness refers to the collection of forces that influence employee retention [12]. High job embeddedness has a multidimensional impact on an individual’s intention to stay with the organization, including improved organizational performance, motivation to perform, and job capabilities [13]. Studies on job embeddedness have reported its correlations with role conflict [5, 10], and job crafting [14].

**Fig. 1:**
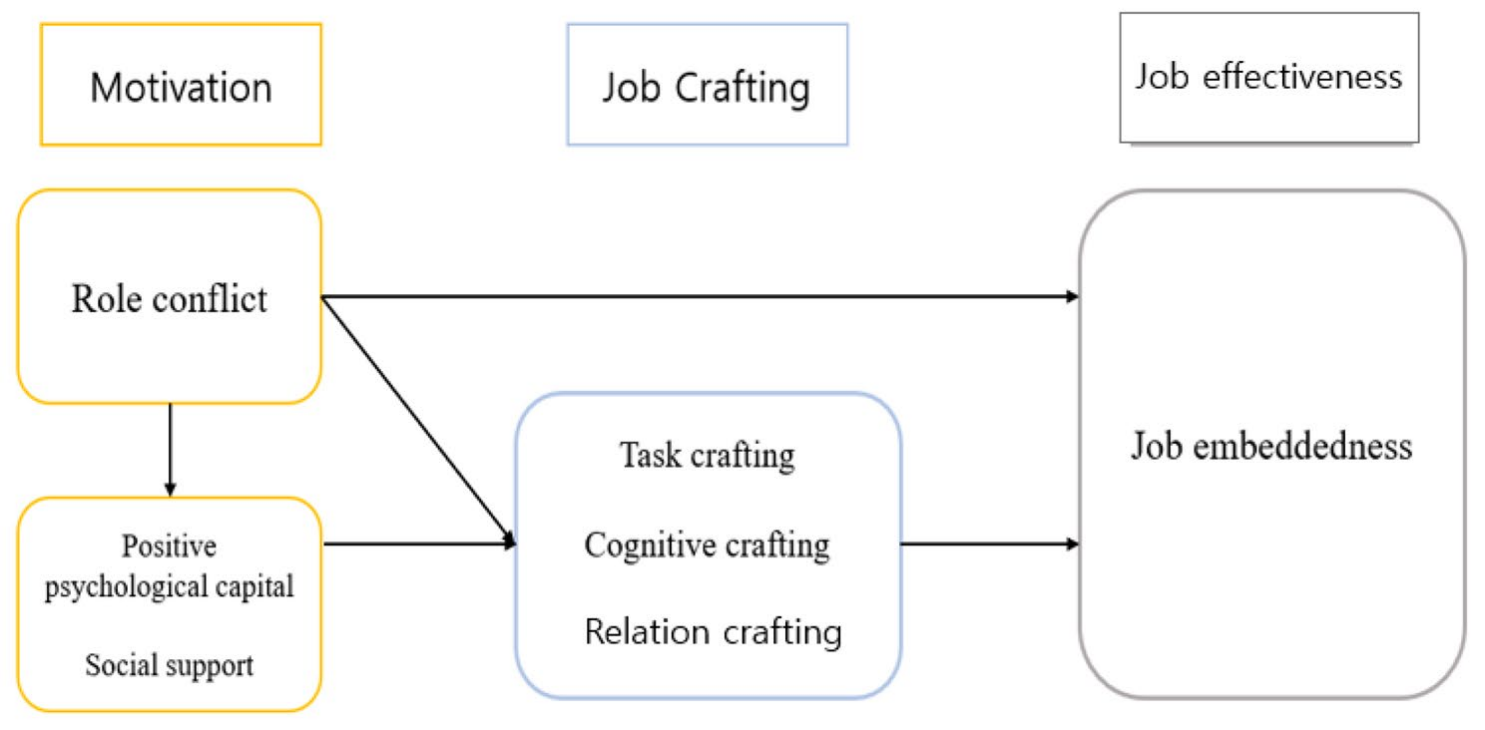
Conceptual framework of our study

Our study aimed to identify the influences that encourage nurses to remain rooted at their place of work—beyond merely staying with the organization—by constructing a structural model of clinical nurses’ job embeddedness based on the job crafting model of Wrzesniewski and Dutton [15] and then testing the fit of the model (Fig. 1).

Motivational factors, as antecedents of the job crafting model, were divided into job control needs, positive self-image, and relationships. We constructed an integrated model including job embeddedness as an outcome of and factor in job effectiveness.

Based on the hypothetical model, we established the following research hypotheses:

Hypothesis 1 (H1). Job crafting has a positive effect on job embeddedness.

Job embeddedness and job crafting have been found to be related [14]. In organizations, it is important to not only recruit talented workers but also ensure they can fulfil their potential [16]. Job crafting has begun to attract attention as a concept of job-related behavior that enables the voluntary and positive performance of one’s job and enhances internal motivation. Originally meaning “force,” “craft” is used in the field of art to mean creating objects through skill and finesse. Job crafting can thus be defined as a process of recreating one’s job, making it more meaningful [15]. The term job crafting assumes that certain job characteristics and attributes motivate people to perform their jobs and that jobs can be changed to become motivating [17]. Job crafting consists of three elements: task crafting, cognitive crafting, and relation crafting [15]. Task crafting refers to the change employees make to either the type or the amount of work they do; cognitive crafting changes the purpose and meaning of work; and relation crafting means building relationships with supervisors or co-workers, or improving them through communication activities with people at work. Antecedents of job crafting include role conflict [5], positive psychological capital [17, 18], and social support [19].

Hypothesis 2 (H2). Role conflict has a negative effect on job embeddedness.

Role conflict affects job embeddedness [14]. Hospitals are specialized and multidisciplinary workplaces providing healthcare services to patients through collaborative efforts [20]. Nurses, who provide nursing care, often experience role conflict owing to heavy workloads, unilateral orders and instructions, lack of autonomy, and limited participation in decision-making [21]. When this role conflict is not addressed, nurses’ turnover intention is likely to be higher, lowering retention intention [22] and increasing turnover rates [23]. That is, role conflict influences job embeddedness.

Hypothesis 3 (H3). Social support has a positive effect on job crafting.

Social support affects job crafting [24]. It is defined as all the positive help that an individual receives through their relationships with others [25] and is a collective term for the mental and physical resources related to an individual’s work that they receive from family, supervisors, co-workers, and others [26]. Social support, as a positive resource that an individual can derive from family and co-workers [25, 27], can reduce tension and promote stability by evoking positive emotions [28]. It thus has a positive impact on job performance and reduces role conflict and the pressure on nurses’ adaptability, while also decreasing their turnover intention [29].

Hypothesis 4 (H4). Positive psychological capital has a positive effect on job crafting.

Positive psychological capital has been studied as a variable to shed light on turnover [30] and highlighted as a key factor of human resource management because it can maximize the potential of individuals [31, 32]. Positive psychological capital refers to a complex, positive psychological state wherein an individual is confident in the success of their performance, optimistic about the present and future, and able to overcome obstacles [33]. Among nurses, positive psychological capital affects their physical and psychological health by reducing negativity and increasing positivity [34], making nurses more innovative and creative in their nursing practice. It also induces positive changes in hospitals by affecting nurses’ job-related attitude and behavior [35].

Hypothesis 5 (H5). Role conflict has a negative effect on positive psychological capital.

Hypothesis 6 (H6). Role conflict has a negative effect on social support.

Hypothesis 7 (H7). Role conflict has a negative effect on job crafting.

In this study, an integrated model of ‘job-related’ factors was constructed, based on the ‘job crafting model’ to identify factors that enable nurses to become embedded in their jobs. It is expected to provide a new understanding of how to effectively manage individual and organizational connections and job engagement, shifting away from the traditional focus on individual nurse turnover. The study aims to provide evidence that can be used to inform effective management of the nursing workforce, thereby contributing to improved quality of care and patient safety. Specifically, this study had the following three aims: first, construct a hypothetical model of the relations among role conflict, positive psychological capital, social support, job crafting, and job embeddedness among clinical nurses; second, test the hypothetical model of clinical nurses’ job embeddedness; and third, establish the relations among role conflict, positive psychological capital, social support, job crafting, and job embeddedness among clinical nurses.

## Methods

### Design

The research design is a descriptive study to examine the relationship between role conflict, positive psychological capital, social support, job crafting and job embeddedness in clinical nurses.

### Participation

Participants were clinical nurses in general and tertiary general hospitals in G province and D metropolitan city. Permission was sought from the directors of nursing at four general hospitals, and a questionnaire explaining the purpose and methodology of the study was distributed to the heads of nursing departments. Consenting nurses completed the questionnaire. Inclusion criteria for the study were nurses who had worked in a general hospital for at least one year and consented to participate in the survey. We excluded new nurses with less than one year of experience and nursing managers. Nurses with less than one year of experience were excluded because they had not yet reached the point of off-the-job education and training to be able to work independently, which may affect their job embeddedness [36].

The criteria for the fulfilment of the structural equation were based on the maximum likelihood (ML) estimation method, which recommends that a minimum sample size of 200‒400 is appropriate for model validation [37]. Considering a 15% dropout rate, we distributed 300 copies of our questionnaire, of which 290 were returned. We used 260 responses in the final analysis after excluding 40 responses that were incomplete or showed an identical pattern in the item responses.

### Tools

#### General characteristics

The general characteristics segment of the questionnaire consisted of seven questions on sex, marital status, age, hospital career, department, position in the organization, and type of duty.

#### Role conflict

We used the scale proposed by Pareek [38], adapted by Kim [39], and modified and supplemented by Son [40]. Permission to use the tool has been granted by the original author. The instrument consists of 22 questions: four for role isolation conflicts, three for role expectation conflicts, three for person–role conflicts, four for role ambiguity conflicts, two for inter-role conflicts, three for role overload conflicts, and three for resource scarcity conflicts. Each question is scored on a Likert scale ranging from 1 = *Not at all* to 5 = *Strongly agree*, with higher scores indicating higher role conflict.

After confirmatory factor analysis, the standardization coefficient should be at least 0.7 to indicate the reliability of the individual observables [41]. In this study, after confirmatory factor analysis, two items that hindered validity were removed, leaving 20 of the 22 items to be analyzed. In the study by Son [40], the Cronbach’s α for reliability was 0.71 to 0.91. In our study, the Cronbach’s α was 0.89.

#### Positive psychological capital

We used the positive psychological capital questionnaire developed by Luthans [33] and modified and supplemented by Yun [42]. The original author has given permission for the tool to be used. The instrument consists of 24 items, including six items each for self-efficacy, hope, resilience, and optimism. Each item is rated on a Likert scale ranging from 1 = *Not at all* to 5 = *Strongly agree*, with higher scores indicating higher levels of positive psychological capital.

After confirmatory factor analysis, we removed two items that hindered validity. In Yun’s study [42], self-efficacy, hope, resilience, and optimism had Cronbach’s α values of 0.86, 0.86, 0.74, and 0.68, respectively. The Cronbach’s α values in our study were 0.89, 0.84, 0.78, and 0.74 for self-efficacy, hope, resilience, and optimism, respectively. The overall Cronbach’s α was 0.89.

#### Social support

We used the instrument developed by House and Wells [43] and modified and supplemented by Jung [44]. Permission to use the tool has been granted by the original author. The instrument consists of 24 questions: eight questions each on support from supervisors, support from co-workers, and support from family and friends. Each question is rated on a Likert scale ranging from 1 = *Not at all* to 5 = *Strongly agree*, with higher scores indicating higher social support. In our study, the Cronbach’s α was 0.93, similar to that in Jung’s study [44].

#### Job crafting

We used the tool developed by Slemp and Vella-Brodrick [45] and adapted to the Korean setting by Lim et al. [46]. The original author has given permission for the tool to be used. The instrument consists of 15 questions: five questions each for task crafting, cognitive crafting, and relation crafting. Each item is rated on a Likert scale ranging from 1 = *Not at all* to 6 = *Strongly agree*. Instrument reliability was Cronbach’s α = 0.91 in the original study [45] and Cronbach’s α = 0.87 in our study.

#### Job embeddedness

We used the tool developed by Mitchell et al. [11] and modified and supplemented by Kim et al. [47]. Permission to use the tool has been granted by the original author. The instrument consists of 18 questions: eight on fit, four on links, and six on sacrifice. Each item is rated on a Likert scale ranging from 1 = *Not at all* to 5 = *Strongly agree*, with higher scores indicating higher job embeddedness. Confirmatory factor analysis showed that one item ‘links’ hindered validity; we removed this item. The Cronbach’s α was.87 in the study by Kim [47] and.88 in our study.

### Data collection process and ethical considerations

This study was approved by the Institutional Review Board of E University (EU21-081) and each questionnaire was approved for use by the researcher. It was conducted from January 10 to February 28, 2022 after obtaining consent from the nursing departments of the study sites. We visited the nursing departments of the hospitals and explained the purpose and content of the study to the department head; after obtaining permission, we distributed questionnaires to each department through the nursing department. We prepared a study manual that explained the purpose of the study, confidentiality guarantee, and rights of the research participant. The explanation included assurances that the data collected would be used for academic purposes only, that there would be no disadvantage as the data would be anonymized, and that absolute confidentiality would be maintained. After reading this manual, the participants voluntarily gave written consent to participate in the study and filled out the questionnaire. Completing the questionnaire took about 15‒20 min. To ensure confidentiality, we asked the participants to seal the questionnaires themselves. We collected the completed questionnaires through the nursing departments. Those participants who completed the questionnaire were offered a small compensation fee.

### Data analysis method

The collected data were analyzed using SPSS 26.0 and AMOS 26.0. We analyzed the general characteristics of the participants and study variables using descriptive statistics. Skewness and kurtosis were examined to verify the normality of the structural equation. Since structural equations are an extension of regression, we checked for multicollinearity before implementing the structural model.

To assess how well the hypothetical model is supported by the data collected, we tested the goodness of fit of the model using ML estimation. To assess the goodness of fit of the structural model, we analyzed the data using the absolute fit index, χ^2^(CMIN), χ^2^/df, standardized root mean square residual (SRMR), root mean square error of approximation (RMSEA), goodness of fit index (GFI), adjusted goodness of fit (AGFI), and comparative fit index (CFI).

We checked the internal consistency of the measures for the latent variables using the average variance extracted (AVE) and construct reliability (CR) values. Discriminant validity refers to the degree of difference between the latent variables and requires low correlation between the measures obtained when measuring a concept. We determined discriminant validity by comparing the respective AVE values between the constructs with the squared correlation coefficient between them [48].

We used modification indices to determine the final model. We selected theoretically relevant models from the order of the largest modification indices and modified the connected models in turn. To test the research hypotheses, we assessed the statistical significance of the indirect and total effects of the model using the bootstrapping method.

## Results

### General characteristics of the participants

The total number of participants in this study was 260, with 90.8% women and 9.2% men; by marital status, 83.5% were single. The mean age of the participants was 28.99 ± 4.86 years, with 51.5% aged 25–29 years. Under one-third had clinical experience of 3 years or less (30.8%) and 30.0% had clinical experience of 5–10 years.

The breakdown by department was as follows: 45.0% general ward, 24.2% intensive care unit, and 12.3% emergency department. The vast majority of the participants were general nurses (91.5%) and 8.5% were charge nurses. In terms of working hours, 88.1% were shift workers and 8.1% were full-time workers. Table 1 shows the general characteristics of the participants.

**Table 1:** General characteristics of the participants (*N* = 260)

Characteristic	Category	N	%	M ± SD
Sex	Male	24	9.2	
	Female	236	90.8	
Marital status	Single	217	83.5	
	Married	43	16.5	
Age (years)	≤ 24	33	12.7	28.99 ± 4.86
	25–29	134	51.5	
	30–34	67	25.8	
	≥ 35	26	10.0	
Hospital career (years)	≤ 2	80	30.8	5.38 ± 4.76
	3–4	68	26.1	
	5–9	78	30.0	
	≥ 10	34	13.1	
Department	General ward	117	45.0	
	ICU	63	24.2	
	OR	10	3.9	
	OBGY/ped	20	7.7	
	ER	32	12.3	
	OPD	18	6.9	
Position	Nurse	238	91.5	
	Charge nurse	22	8.5	
Type of duty	Three-shift duty	228	87.7	
	D/E fixed	6	2.3	
	Regular work	22	8.5	
	Other	4	1.5	

Note: SD = Standard Deviation; ICU = Intensive Care Unit; OR = Operation Room; OBGY = Obstetric Gynecology; Ped = Pediatrics; ER = Emergency Room; OPD = Outpatient Department; D/E = Day or Evening

### Descriptive statistics and normality checks

To determine whether the data met the assumption of normality, we measured the skewness and kurtosis of the measured variables and found that their absolute values did not exceed 2. Hence, they all met the assumption of normality. The confirmatory factor analysis of the variables showed that the AVE was more than 0.7 and CR was more than 0.9, confirming convergent validity. An AVE of 0.5 or higher and CR of 0.7 or higher are considered to indicate meaningful convergent validity [48].

To verify discriminant validity, we checked the correlations among the variables. The highest correlation was between positive psychological capital and job embeddedness (0.776, square of 0.602). The AVE of positive psychological capital was 0.756 and that of job embeddedness was 0.701, both greater than the squared value of the correlation coefficient, indicating discriminant validity. Table 2 shows the descriptive statistics and normality checks for the metrics.

**Table 2:** Descriptive statistics and normality checks (*N* = 260)

Variable	Mean	SD	Skewness	Kurtosis	AVE	CR
**Role conflict**	2.57	0.51	− 0.17	− 0.23	0.732	0.997
Role isolation	2.12	0.53	0.26	0.17		
Role expectation	2.40	0.81	0.25	− 0.42		
Person–role	2.68	0.79	0.17	− 0.51		
Role ambiguity	1.71	0.63	0.67	0.63		
Inter-role	2.63	0.91	0.10	− 0.64		
Role overload	3.02	0.86	− 0.18	− 0.24		
Resource scarcity	3.35	0.85	− 0.36	− 0.10		
**Positive psychological capital**	3.19	0.50	− 0.03	1.11	0.756	0.996
Self-efficacy	2.83	0.73	0.14	0.34		
Hope	3.52	0.58	0.29	1.34		
Optimism	3.13	0.64	− 0.15	0.00		
Resilience	3.29	0.62	− 0.13	0.42		
**Social support**	3.73	0.55	0.00	− 0.04	0.736	0.997
Support from supervisors	3.57	0.70	− 0.40	0.43		
Support from co-workers	3.90	0.58	− 0.30	0.27		
Support from family and friends	3.74	0.72	− 0.26	− 0.02		
**Job crafting**	3.95	0.68	− 0.25	0.10	0.724	0.992
Task crafting	3.90	0.75	− 0.19	0.11		
Cognitive crafting	3.97	0.94	− 0.28	− 0.21		
Relation crafting	3.97	0.77	− 0.09	0.59		
**Job embeddedness**	3.07	0.50	0.60	0.84	0.701	0.994
Fit	3.30	0.62	− 0.38	0.75		
Sacrifice	2.36	0.63	− 0.09	− 0.19		

Note: SD = Standard Deviation; AVE = Average Variance Extracted; CR = Construct Reliability

### Hypothetical model and modification

In this study, all the correlation coefficients among the measured variables were less than 0.7 compared with a recommended threshold of 0.5, while the variance inflation factors were less than 1.8, meaning no problem with multicollinearity. We tested the goodness of fit of the model using ML estimation to assess how well the hypothetical model of job embeddedness is supported by the data collected. The model fit was 2.889 for CMIN/df, 0.868 for GFI, 0.822 for AGFI, 0.879 for CFI, and 0.085 for RMSEA. The model was modified by selecting the theoretically relevant models with the largest modification indices to improve the model fit and then connecting them to determine the final modified model. The final model had a CMIN/df of 2.617, GFI of 0.884, AGFI of 0.840, CFI of 0.899, and RMSEA of 0.079, showing that the fit had improved (Table 3).

**Table 3:** Goodness of fit of the model and modified model

Model	CMIN			CMIN/df	GFI	AGFI	CFI	SRMR	RMSEA
	χ^2^	df	*p*						
Reference			> 0.05	≤ 3	≥ 0.9	≥ 0.8	≥ 0.9	≤ 0.08	≤ 0.10
Hypothetical model	326.448111	113	< 0.001	2.889	0.868	0.822	0.879	0.089	0.085
Modified model	290.442111	111	< 0.001	2.617	0.884	0.840	0.899	0.076	0.079

Note: GFI = Goodness of Fit Index; AGFI = Adjusted Goodness of Fit Index; CFI = Comparative Fit Index; SRMR = Standardized Root Mean Square Residual; RMSEA = Root Mean Square Error of Approximation

### Pathways of the model

**Fig. 2:**
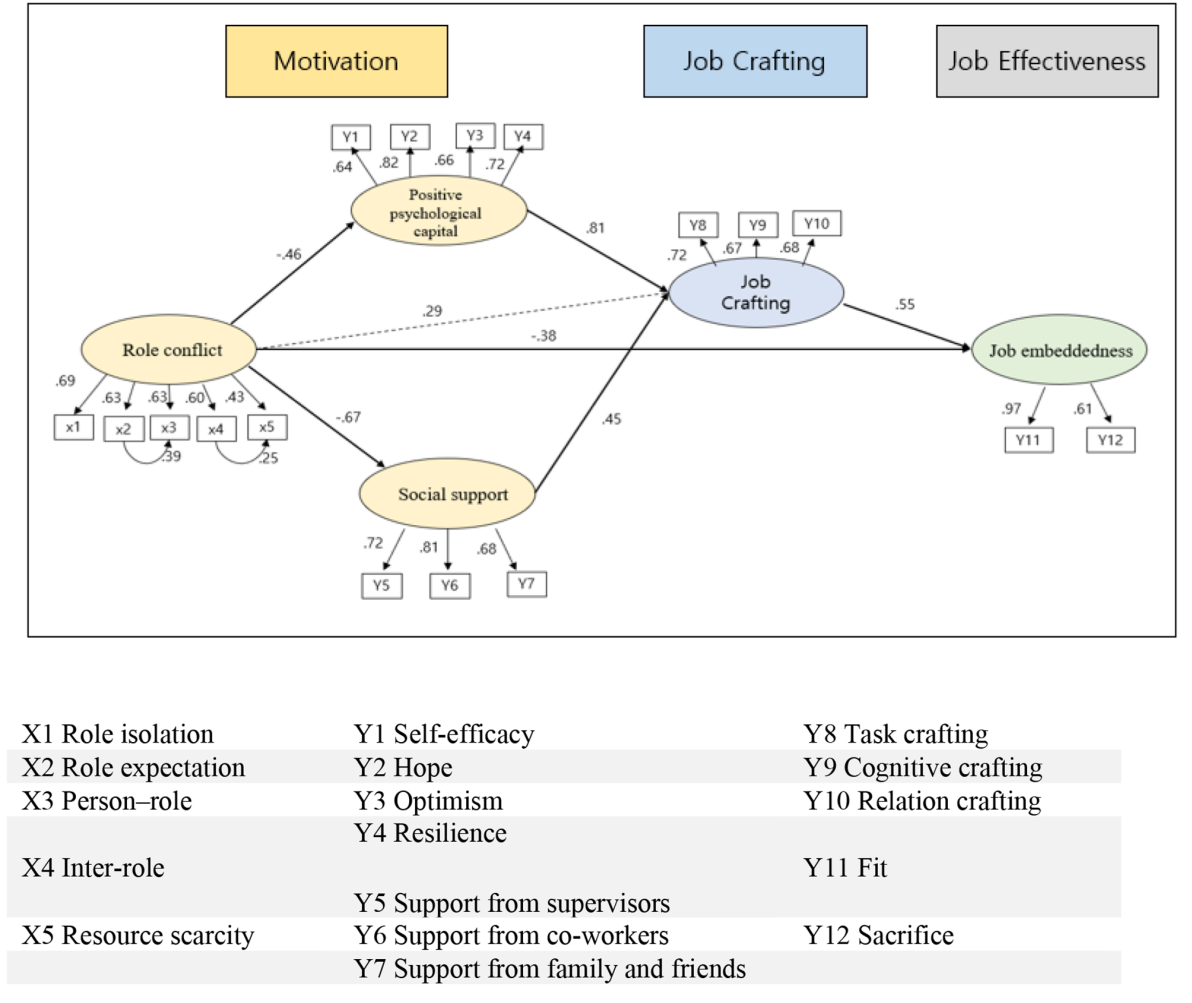
Path diagram of the modified model

The effect of role conflict on positive psychological capital (γ = − 0.584, *p* <.001) was statistically significant, with an explanatory power of 21.1%. The effect of role conflict on social support (γ = − 0.891, *p* <.001) was statistically significant, with an explanatory power of 44.5%. Job crafting was statistically significantly affected by role conflict (γ = 0.430, *p* =.009), positive psychological capital (β = 0.933, *p* <.001), and social support (β = 0.496 *p* <.001), with an explanatory power of 76.9%. Job embeddedness was statistically significantly affected by role conflict (γ = − 0.624, *p* <.001) and job crafting (β = 0.609, *p* <.001), with an explanatory power of 60.4%. The paths of the modified model are shown in Table 4; Fig. 2.

**Table 4:** Pathways of the models

Endogenous variables $\leftarrow$ Exogenous Variables	RW	SRW	SE	CR	*p*	SMC
Positive psychological capital						
$\leftarrow$ Role conflict	− 0.584	− 0.460	0.111	-5.276	< 0.001	0.211
Social support						0.445
$\leftarrow$ Role conflict	− 0.891	− 0.667	0.127	-7.011	< 0.001	
Job crafting						
$\leftarrow$ Role conflict	0.430	0.228	0.165	2.610	0.009	0.769
$\leftarrow$ Positive psychological capital	0.933	0.806	0.121	7.705	< 0.001	
$\leftarrow$ Social support	0.496	0.431	0.112	4.421		
Job embeddedness						
$\leftarrow$ Role conflict	− 0.624	− 0.382	0.112	-5.556	< 0.001	0.604
$\leftarrow$ Job crafting	0.609	0.548	0.078	7.829	< 0.001	

Note: RW = Regression Weight; SRW = Standardized Regression Weight; SE = Standard Error; CR = Critical Ratio, SMC = Squared Multiple Correlations

### Verification of the research hypotheses

To test the research hypotheses, we analyzed the direct, indirect, and total effects among the latent variables. The bootstrapping method was used to verify the statistical significance of these effects (Table 5).

**Table 5:** Direct, indirect, and total effects of the modified model

Endogenous variables $\leftarrow$ Exogenous variables	Standardized direct effects(p)	Standardized indirect effects(p)	Standardized total effects(p)
Positive psychological capital			
$\leftarrow$ Role conflict	− 0.460(0.005)		− 0.460(0.005)
Social support			
$\leftarrow$ Role conflict	− 0.667(0.006		− 0.667(0.006)
Job crafting			
$\leftarrow$ Role conflict	0.292(0.055)	− 0.671(0.003)	− 0.379(0.008)
$\leftarrow$ Positive psychological capital	0.806(0.007)		0.806(0.007)
$\leftarrow$ Social support	0.451(0.004)		0.451(0.004)
Job embeddedness			
$\leftarrow$ Role conflict	− 0.382(0.007)	− 0.208(0.003)	− 0.589(0.006)
$\leftarrow$ Job crafting	0.548(0.005)		0.548(0.005)
$\leftarrow$ Positive psychological capital		0.441(0.005)	0.441(0.005)
$\leftarrow$ Social support		0.247(0.003)	0.247(0.003)

The direct (γ = − 0.460, *p* =.005) and total (γ = − 0.460, *p* =.005) effects of role conflict on positive psychological capital were significant. Similarly, the direct (γ = − 0.667, *p* =.006) and total (γ = − 0.667, *p* =.006) effects of role conflict on social support were significant. Therefore, H5 and H6 were supported. Meanwhile, the direct (γ = 0.292, *p* =.055), indirect (γ = − 0.671, *p* =.003), and total (γ = − 0.379, *p* =.008) effects of role conflict on job crafting were significant. Therefore, H7 was rejected.

The results also showed support for H1 to H4. The direct (β = 0.806, *p* =.007) and total (β = 0.806, *p* =.007) effects of positive psychological capital on job crafting were significant. The direct (β = 0.451, *p* =.004) and total (β = 0.451, *p* =.004) effects of social support on job crafting were significant. The direct (γ = − 0.382, *p* =.007), indirect (γ = − 0.208 *p* =.003), and total (γ = − 0.589, *p* =.006) effects of role conflict on job embeddedness were significant. Lastly, the direct (β = 0.548, *p* =.005) and total (β = 0.548, *p* =.005) effects of job crafting on job embeddedness were significant.

## Discussion

This study found that job crafting and role conflict directly affected job embeddedness, with job crafting having the strongest effect. These findings are supported by a previous study that showed a correlation between job crafting and job embeddedness [14]. Higher job crafting relates to higher job embeddedness, which directly affects job effectiveness [15]. This is because job crafting affects the organization by employees changing the methods or boundaries of the job [15], thereby increasing the meaning of and satisfaction with work by changing its scope based on changes in nursing behavior and work perception [49]. Tims and Bakker [50] argued that job crafting is more active when autonomy or task independence is highly guaranteed. That is, organizational members must have job autonomy to be more actively involved in job crafting, which leads them to assign meaning to their jobs and set their own goals [51]. In addition, job crafting is increased through motivation and positive interactions with co-workers [50, 52]. Job crafting involves members transforming their own jobs. In nursing, this process becomes a form of creative work where the job is adapted to the needs of the patient, ultimately improving the patient’s wellbeing. Therefore, increased job embeddedness is promoted by job crafting, for which it is crucial to increase job autonomy, meaningful work, and positive interactions with co-workers. By experiencing this process, nurses become embedded in the organization.

The present study found that social support had a direct effect on job crafting and an indirect effect on job embeddedness. These findings are consistent with previous research showing that social support is related to job crafting [24] and also affects job embeddedness [53]. Relation crafting increases collaborations and interactions with superiors and colleagues from other departments through active exchanges [54]. As nursing is a team-oriented profession, support from co-workers can reduce emotional tension in tense situations [55], which in turn affects job crafting. Moreover, support from superiors—as feedback, shared information, or counseling on organizational life—has a huge impact on employees [56]. When supervisors understand and acknowledge employees’ perspectives, employees are provided with possible options [57], motivated to do their jobs, and more effective. Thus, job crafting involves not only actively engaging in work but also building and improving relationships [58]. Therefore, it’s important to create an organizational culture where nurses can receive positive feedback from colleagues and managers as they strive to change their roles to deliver personalized care to patients.

This study found that positive psychological capital had direct and indirect effects on job embeddedness, consistent with the findings of previous studies [17, 59]. As positive psychological capital influences employees’ positivity toward their work and the organization [60], individuals with a highly positive mentality tend to work actively toward achieving their goals and take the initiative in designing their work [61]. They try to overcome difficulties patiently by using accessible resources based on their optimistic perspective of a situation [33]. The effect of positive psychological capital on job embeddedness was indirect via job crafting. Even when challenging situations arise in nursing, positive thinking can improve job performance, leading to higher job satisfaction [62], which results in greater engagement at work and ultimately a greater intention to stay with the organization [63, 64]. High positive psychological capital is a crucial factor in job embeddedness and is associated with nurses’ performance at work and therefore their successful achievement of organizational goals [65]. When managers understand and recognize nurses’ perspectives and provide information about the options available, it motivates staff to do their jobs effectively.

Meanwhile, role conflict had a direct negative effect on job embeddedness. Although research confirming the relation between role conflict and job embeddedness in clinical nurses is lacking, a study on nurses working in nursing homes reported that role conflict has a negative effect on job embeddedness [66], supporting the findings of our study. Nurses face conflicts when they interact with various people, which is an unavoidable part of providing nursing care [67]. Given that high role conflict is associated with increased negative emotions, low self-esteem [68], and low organizational commitment, which raise turnover intention [69], organizations should seek to moderate the role conflict of employees to increase their job embeddedness.

Role conflict directly positively affected job crafting and negatively affected job embeddedness. Pareek [38] categorized the types of role conflicts as role isolation conflicts, role expectation conflicts, person–role conflicts, role ambiguity conflicts, etc. suggesting that various factors cause role conflicts. Role conflict and job crafting have several positive impacts. Role conflict occurs under conditions of low autonomy to change one’s scope of work, which leads to an increase in job crafting to create a new job from a given job [52]. Some types of role conflicts may also arise in the process of taking the initiative and being creative. When role conflict is not resolved, job embeddedness is reduced. However, the causal relation between role conflict and job crafting could not be confirmed in our cross-sectional study with unknown time points. Future research could thus study the effect of role conflict on job crafting more in depth to bridge this research gap.

Indeed, our study has a number of limitations that should be acknowledged. First, since this is a cross-sectional study aiming to build a model of job embeddedness based on the theory, temporal causality could not be confirmed. Second, this study is limited to general and tertiary general hospitals. Given the various types of hospitals, it may be difficult to extend the findings to all hospitals. Nevertheless, this study is significant, as it provides a basis for nursing research on job embeddedness despite the lack of studies on the antecedents of job embeddedness, by building a hypothetical model of job embeddedness among clinical nurses and identifying the influencing factors. The implications of these findings are as follows. Job crafting is a form of creative work in the nursing process that adapts tasks to patient needs and ultimately improves patient well-being. As nurses strive to creatively transform their work, they may experience conflict with their existing roles. Managers need to facilitate the conflict process by understanding and acknowledging nurses’ perspectives and providing information about available options. This will motivate nurses to do their jobs effectively. Job autonomy, meaningful work and positive interactions with colleagues can therefore increase job embeddedness through job crafting.

## Conclusions

This study used a model-building research approach to analyze the factors affecting clinical nurses’ job embeddedness and explored the pathways to increase their embeddedness, thereby providing a basis for efficient human resource management. Job crafting affects job embeddedness when positive psychological capital and social support are high. When job crafting takes place, role conflict increases, and if job crafting becomes difficult because of severe role conflict, job embeddedness decreases. Hence, role conflict and job crafting directly explain over 60% of the job embeddedness of clinical nurses.

To increase the job embeddedness of nurses, hospitals need to enhance nurses’ job autonomy. Nurses also need organizational support to enhance positive psychological capital and social support. This should be followed by enhanced training and programs to strengthen them. All these can ultimately contribute to efficient human resource management in hospitals.

## Data Availability

Data available upon request by email to the corresponding author.
